# *Brucella abortus* Choloylglycine Hydrolase Affects Cell Envelope Composition and Host Cell Internalization

**DOI:** 10.1371/journal.pone.0028480

**Published:** 2011-12-08

**Authors:** María Inés Marchesini, Joseph Connolly, María Victoria Delpino, Pablo C. Baldi, Cesar V. Mujer, Vito G. DelVecchio, Diego J. Comerci

**Affiliations:** 1 Instituto de Investigaciones Biotecnológicas-Instituto Tecnológico de Chascomús, Universidad Nacional de San Martín, Consejo Nacional de Investigaciones Científicas y Técnicas, Buenos Aires, Argentina; 2 Vital Probes, Inc., Mayfield, Pennsylvania, United States of America; 3 Instituto de Estudios de la Inmunidad Humoral, Facultad de Farmacia y Bioquıímica, Universidad de Buenos Aires, Consejo Nacional de Investigaciones Científicas y Técnicas, Buenos Aires, Argentina; National Council of Sciences (CONICET), Argentina

## Abstract

Choloylglycine hydrolase (CGH, E.C. 3.5.1.24) is a conjugated bile salt hydrolase that catalyses the hydrolysis of the amide bond in conjugated bile acids. Bile salt hydrolases are expressed by gastrointestinal bacteria, and they presumably decrease the toxicity of host's conjugated bile salts. *Brucella* species are the causative agents of brucellosis, a disease affecting livestock and humans. CGH confers *Brucella* the ability to deconjugate and resist the antimicrobial action of bile salts, contributing to the establishment of a successful infection through the oral route in mice. Additionally, *cgh*-deletion mutant was also attenuated in intraperitoneally inoculated mice, which suggests that CGH may play a role during systemic infection other than hydrolyzing conjugated bile acids. To understand the role CGH plays in *B. abortus* virulence, we infected phagocytic and epithelial cells with a *cgh*-deletion mutant (*Δcgh)* and found that it is defective in the internalization process. This defect along with the increased resistance of *Δcgh* to the antimicrobial action of polymyxin B, prompted an analysis of the cell envelope of this mutant. Two-dimensional electrophoretic profiles of *Δcgh* cell envelope-associated proteins showed an altered expression of Omp2b and different members of the Omp25/31 family. These results were confirmed by Western blot analysis with monoclonal antibodies. Altogether, the results indicate that *Brucella* CGH not only participates in deconjugation of bile salts but also affects overall membrane composition and host cell internalization.

## Introduction

Bile acids are synthesized from cholesterol in hepatocytes. Prior to being exported from the liver, bile acids are conjugated by an amide bond to taurine or glycine to produce bile salts. In addition to their lipid-emulsifying function in the intestinal tract, bile acids serve to control bacterial overgrowth in the small intestine. Given their antimicrobial action, it has been proposed that intestinal microbiota has evolved a system that reduces the detergent properties of bile salts, promoting the survival and colonization of bacteria in the gut [1]. Bacterial metabolism of conjugated bile acids is initiated by bile salt hydrolase (E.C. 3.5.1.24), also referred to as choloylglycine hydrolase (CGH), which catalyzes the hydrolysis of amide bonds of conjugated bile acids, resulting in the release of free primary bile acids and amino acids.

Genes coding for CGH were identified in *Brucella* genomes [2]. They are highly conserved in all sequenced *Brucella* species, and multiple alignment analysis revealed that residues at the active site are highly conserved [2]. *Brucella* species are intracellular pathogens responsible for brucellosis, a worldwide distributed zoonosis. Pathogenic *Brucella* mainly infect cattle, swine, goats, sheep and dogs, causing abortion in females and sterility in males [3]. Although *Brucella* species do not reside in the gut of infected mammals, oral infection is one of the entry routes either through consumption of contaminated dairy products or contact with infected placental tissues [4]. Recently, we demonstrated that *B. abortus* CGH can deconjugate bile salts and that this enzymatic activity enhances *Brucella* survival in a bile-containing environment [2]. It was also observed that a *cgh*-deletion mutant is attenuated in intragastrically infected mice, demonstrating that CGH may help *Brucella* to resist the detergent action of bile salts upon oral route entry. The *cgh*-deletion mutant was also attenuated in intraperitoneally inoculated mice; suggesting that CGH may be involved in activities other than hydrolysis of conjugated bile acids and may play a role during systemic infection. Interestingly, CGH has also been identified as a component of the *Brucella* containing vacuole (BCV), a membrane-bound compartment that contains the bacterium during its intracellular life cycle [5], reinforcing the idea that the enzyme could be important for these stages.

In this work, we demonstrate that *B. abortus* CGH mutant has several pleiotropic defects related to an altered membrane function and composition such as faster generation time during both vegetative and intracellular growth, resistance to polymyxin B, differential expression profile of several major outer membrane proteins and a defect in cellular adhesion and internalization in phagocytic and non-phagocytic cells. All these defects strongly suggest that CGH, besides its role as a bile-salt deconjugating enzyme, plays and important and yet uncharacterized function related to the structure and composition of the *Brucella* cell envelope.

## Materials and Methods

### Bacterial strains and growth conditions

Bacterial strains used in this study are: smooth virulent wild-type *Brucella abortus* strain 2308 (S2308); unmarked deletion mutant *Δcgh* (BAB1_1488) [2]; complemented *Δcgh* mutant strain [2]; S2308 pGFP [6]; and *Δcgh* pGFP. *B. abortus* strains were grown in tryptic soy agar (TSA) or in tryptic soy broth (TSB) (Difco/Becton-Dickinson, Sparks, MD) at 37°C on a rotary shaker for 16−20 h. Media acidification (pH 5.5) was achieved by addition of citrate buffer to the growth media. Growth was monitored by measuring the optical density of the cultures at 600 nm (OD_600_). When indicated, media were supplemented with 50 µg/ml kanamycin, 50 µg/ml ampicillin and/or 5 µg/ml nalidixic acid. All work with live *B. abortus* was performed in a biosafety level 3 laboratory facility at University of San Martín. *Escherichia coli* strain S17.1 (λpir) was grown in Luria Broth (LB) at 37°C with 50 µg/ml kanamycin.

### Construction of strain Δcgh pGFP

pGFP [6] was introduced in strain *Δcgh* by biparental mating as described in [6].

### Assessment of B. abortus resistance to bovine bile and polymyxin B

Wild-type *B. abortus* S2308 and *Δcgh* mutant strains were grown in TSB with antibiotic and harvested at late exponential phase. Bacterial pellets were washed twice with TSB and resuspended to an OD_600_ of 1 in TSB. Growth inhibition was evaluated by colony forming units (CFU) counts determined by plating serial dilutions on TSA supplemented with the indicated concentrations of bovine bile or polymyxin B.

### Bacterial infection and replication assays

The human epithelial cell line HeLa and the murine macrophage cell line J774.A1 (purchased from American Type Culture Collection, ATCC) were used. Cell lines were maintained and plated as previously described [7]. Cells (5×10^4^/well) were seeded on 24-well plates in media without antibiotics 24 h before infection. *B. abortus* infections were carried out at the indicated multiplicity of infection (MOI). Bacteria were centrifuged onto cells at 400×*g* for 10 min. After 30 min (J774.A1 cells) or 60 min (HeLa cells) wells were gently washed three times with phosphate-buffered saline (PBS) and incubated for 60 min with fresh medium containing 50 µg/ml gentamicin and 100 µg/ml streptomycin to kill noninternalized bacteria. Thereafter, antibiotics concentrations were decreased to 10 µg/ml gentamicin and 20 µg/ml streptomycin. At the indicated times, infected cells were either washed three times with PBS and lysed with 500 µl 0.1% Triton X-100 in PBS (Sigma-Aldrich) or processed for immunoflourescence staining as described below. The intracellular CFU counts were determined by plating serial dilutions of cell lysates on TSA with the appropriate antibiotic.

### Immunofluorescence microscopy

J774.A1 macrophagic cells were plated on glass coverslips and infected as described above but without the addition of antibiotics to prevent killing of extracellular bacteria. At the indicated times, coverslips were washed with PBS and cells were fixed for 15 min in 3% paraformaldehyde (pH 7.4) at 37°C. Coverslips were then processed for immunofluorescence labeling as previously described [8]. The primary antibodies used for immunofluorescence microscopy were rabbit anti-*Brucella* polyclonal antibody (1∶1,500); M84 mouse anti-*Brucella* OPS monoclonal antibody (1∶1,000); rat anti-mouse LAMP-1 1D4B monoclonal antibody (1∶400) (Developmental Studies Hybridoma Bank, Department of Biological Sciences, University of Iowa) and rabbit polyclonal anti-Calnexin (1∶1,000) (Sigma). The secondary antibodies used were Alexa Fluor 568 goat anti-rabbit and Alexa Fluor 488 rat anti-mouse (1∶5,000) (Molecular Probes Invitrogen Co.). For DNA staining, Hoechst dye at 2 µg/ml was used. After labeling, coverslips were mounted onto slides with FluorSave (Calbiochem). Samples were examined on a Nikon microscope (Eclipse E600). Images of 1024×1024 pixels were then assembled using Adobe Photoshop CS.

### Western blot analysis

To monitor the expression levels of outer membrane proteins in *B. abortus* strains, bacteria were grown in TSB and harvested at stationary phase. Equivalent bacterial pellets were resuspended in Laemmli buffer and samples were subjected to SDS-PAGE. Proteins were transferred onto nitrocellulose membranes using a semi-dry transfer procedure. Immunobloting was performed using mouse monoclonal antibodies (kindly provided by Dr. Axel Cloeckaert) against Omp25 (A59/05F01/C09), Omp2b (A63/05A07/A08), Omp31 (A59/10F09/G10), Omp1 (A53/10B02/A01) and Omp19 (A76/18B02/D06).

### Preparation of cell envelope-associated proteins

Preparation of total membranes of *B. abortus* strains was carried out as described previously [9]. Total protein concentration was determined using the Bio-Rad (Hercules, CA) protein stain with bovine serum albumin (BSA) as a standard. All samples, including the BSA standards were dissolved in C7 resuspension buffer.

### Two-dimensional gel electrophoresis (2DE)

All 2DE analyses were carried out with the ElectrophoretIQ^3^ system (Proteome Systems). Supplies and reagents for 2DE were purchased from Proteome Systems and used according to manufacturer's instructions. Two hundred µl (30 µg) of membrane protein mixture was separated by IEF on 11 cm (pH 4 to 7) linear immobilized pH gradient (IPG) strips. After 4 hours of rehydration, the following focusing parameters were applied: 50 µA per strip, linear voltage increase over 8 hours from 100 V to 10,000 V, and finally 10,000 V for 8 hours. After IEF, IPG strips were equilibrated in Equilibration Buffer and applied onto a 6−15% gradient SDS polyacrylamide gel. Gels were electrophoresed for 1.5 hours at 300 mA and 500V, washed in 10% acetic acid/20% methanol, and stained with ProteomIQ Blue.

### Gel Analysis

The experimental pI and Mr values of each protein were determined using the 2D Phoretix program by Nonlinear Dynamics (Newcastle upon Tyne, UK). Precision Protein Standards Plug purchased from Bio-Rad (Hercules, CA) was included during electrophoresis as molecular weight standards. Each sample was run in triplicate and an average gel was generated using the 2D Phoretix software. Spots present in at least two of the three subgels were included in the average gel.

### In-gel trypsin digestion and MALDI-TOF MS

Protein spots were excised from the 2DE gels using the Xcise robotic workstation (Proteome Systems). Gel plugs were sequentially washed with 50 mM ammonium bicarbonate and 50% acetonitrile, dried, and treated with 1.6 mg/ml of trypsin in 50 mM ammonium bicarbonate at 37°C overnight. Tryptic peptides were applied to MALDI-TOF MS target plate in a solution of 10 mg/ml α-cyano-4-hydroxycinnamic acid in 0.1% trifluoroacetic acid and 50% acetonitrile. MS spectra (100 profiles per spectrum) were obtained using an Axima-CFR plus (Shimadzu Biotech, Woburn, MA) in a positive ion reflectron mode with a source voltage of 25,000 V and a laser intensity of 55%. Peptide mass fingerprints were analyzed and searched against the theoretical spectra of *B. melitensis* 16 M or *B. abortus* S2308 using the Mascot Daemon software package (Matrix Science, London). The search parameters were: maximum of one missed cleavage by trypsin; fixed modification of oxidized methionine; charged state of +1; and mass tolerance of ±0.5 Da.

## Results

### In vitro characterization of B. abortus Δcgh deletion mutant

**Figure 1:**
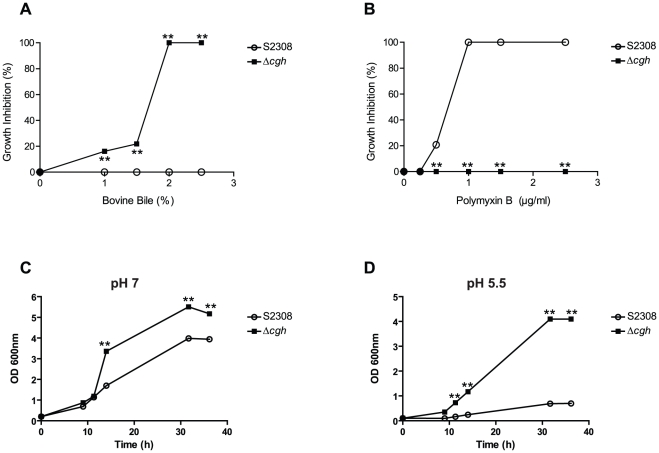
In vitro characterization of B. abortus Δcgh deletion mutant. Growth inhibition of wild-type *B. abortus* (S2308) and its isogenic deletion mutant *Δcgh* in TSA plates containing different concentrations of bovine bile (**A**) and polymyxin B (**B**). Each determination is the mean ± SD of two independent experiments performed in duplicate and indicate the percentage of bacterial growth inhibition relative to the number of viable bacteria in TSA medium without the addition of bovine bile or polymyxin B. *B. abortus* (S2308) and its isogenic deletion mutant *Δcgh* were grown in TSB pH 7 (**C**) or pH 5.5 (**D**). At the indicated times, growth was monitored by measuring the OD_600_ of the cultures. Each determination is the mean ± SD of two independent experiments performed in duplicate. Statistical analysis was performed with a *t* test. *, *P*<0.05; **, *P*<0.01 (compared with strain S2308).

As revealed by a previous study, *B. abortus* CGH deconjugates bile salts and confers resistance to their antimicrobial action [2]. Consistent with these results, the growth of *B. abortus Δcgh* deletion mutant was impaired by bovine bile in a dose dependent manner, while growth of wild-type strain 2308 remained unaffected in the presence of 2.5% bile (Fig. 1A). To determine whether this increased sensitivity to bile salts may be a marker of a generalized sensitivity of *Δcgh* to substances with detergent action, we next assessed growth inhibition of wild-type and mutant strains grown in plates containing different concentrations of polymyxin B, a polycationic bactericidal agent with a detergent-like mode of action [10]. Contrary to the results obtained with bovine bile, growth of wild-type strain 2308 was completely impaired in presence of 1% polymyxin B, while *Δcgh* growth remained unaltered at 2.5% polymyxin B (Fig. 1B). Additionally, *Δcgh* growth impairment was also assessed in media containing either 1% sarkosyl, triton X-100, SDS or zwittergent. In contrast to the results obtained with bovine bile and polymyxin B, the mutant strain behaved like the wild-type strain (not shown), indicating that the lack of CGH only affects *B. abortus* resistance to the detergent action of bile salts. Sensitivity to bovine bile and resistance to polymyxin B were not related to the integrity of the lipopolysaccharide (LPS) since no differences between the wild-type and the mutant strain were observed by Western blot analysis with monoclonal antibodies against *Brucella* rough and smooth LPS (not shown).

Next, we evaluated the growth performance of the mutant in comparison to wild-type *B. abortus* S2308. As shown in Fig. 1C and D, *Δcgh* exhibited a significant faster growth rate than S2308 both at pH 7 and 5.5, as well as an increased resistance to acidic pH. Taken together, these results indicate that absence of *B. abortus* CGH results in sensitivity to bile salts, increased resistance to the cationic detergent polymyxin B, and a faster growth rate even under acidic conditions. The pleiotropic effects observed in *Δcgh* suggest that, besides its bile salt deconjugating activity, CGH plays an important role in *B. abortus* physiology.

### B. abortus Δcgh is defective in adhesion and cellular internalization

**Figure 2:**
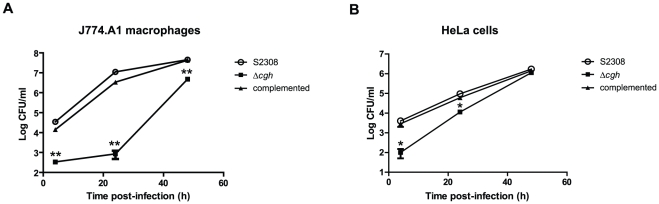
*B. abortus Δcgh* exhibits lower CFU counts during the early infection stages than the wild-type S2308. Intracellular replication of wild-type *B. abortus* (S2308), its isogenic deletion mutant (*Δcgh*) and the complemented mutant in J774.A1 macrophages (**A**) and HeLa cells (**B**). Monolayers of J774A.1 cells (MOI 10∶1) or HeLa cells (MOI 100∶1) were inoculated as described in the text and CFU counts were determined at the indicated times. Each determination is the mean ± SD of two independent experiments performed in duplicate. Statistical analysis was performed with a *t* test. *, *P*<0.05; **, *P*<0.01 (compared with strain S2308).

Previous studies demonstrated that *Δcgh* mutant is attenuated in mice infected through both the oral and intraperitoneal routes [2]. To test whether *Δcgh* is also deficient in its ability to invade and replicate intracellularly, HeLa epithelial cells and J774.A1 macrophages were infected with the wild-type S2308, the *Δcgh* mutant or its complemented strain. The numbers of intracellular bacteria were scored at different times post-infection (p.i.). As shown in Fig. 2A, *Δcgh* showed a significant decrease in the intracellular CFU counts in comparison to the wild type (2 log_10_ units at 4 h p.i. *P = 0.0012*; 4.2 log_10_ units at 24 h p.i. *P = 0.0024*). Afterward, the CFU increased exponentially with a higher growth rate than that of the wild type, thus indicating that the small fraction of internalized *Δcgh* were able to replicate. However, at 48 h p.i., the intracellular CFU of *Δcgh* remained significantly lower than that of the wild type (1 log_10_ units at 48 h p.i. *P = 0.0030*). As expected, the complemented strain behaved like the wild-type strain during the time-course of the experiment, exhibiting similar numbers of CFUs retrieved from infected cells (Fig. 2A). When HeLa cells were infected, a similar, but less drastic phenotype was also observed for the *Δcgh* mutant (Fig. 2B).

**Figure 3:**
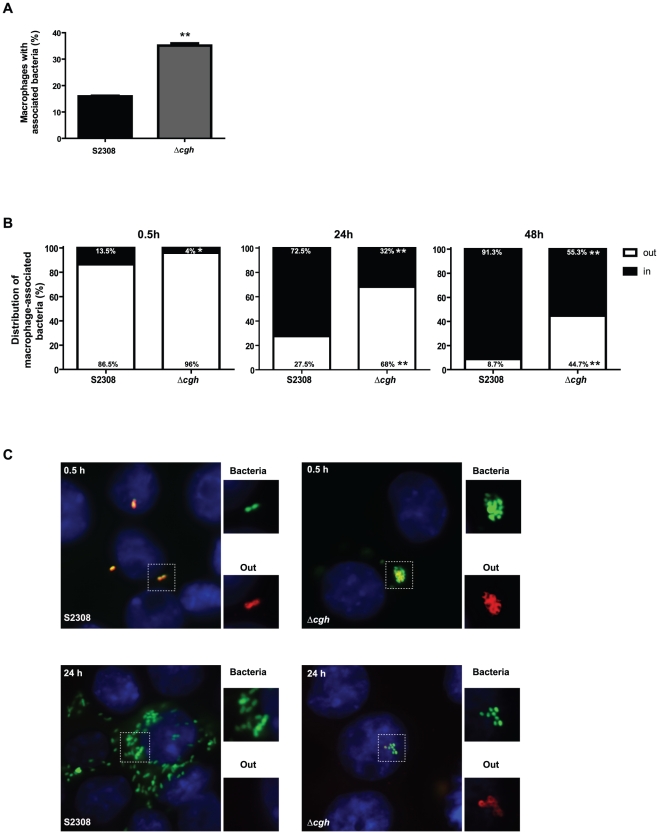
*B. abortus Δcgh* is defective in cellular adhesion and internalization. (**A**) Quantification of J774.A1 macrophages with associated bacteria (*B. abortus* S2308 pGFP or *Δcgh* pGFP) at 0.5 h p.i. (MOI 10∶1). Each determination is the mean ± SD of two independent experiments performed in duplicate. Statistical analysis was performed with a *t* test. *, *P*<0.05; **, *P*<0.01 (compared with strain S2308). (**B**) Quantification of the distribution (intracellular or extracellular) of bacteria per infected cell (J774.A1) at 0.5, 24 and 48 h after infection (MOI 10∶1). Total bacteria (intracellular and extracellular) were visualized in green (*B. abortus* S2308 pGFP or *Δcgh* pGFP), while extracellular bacteria were stained using a rabbit polyclonal antibody against *Brucella* and a secondary antibody conjugated to Alexa Fluor 568 (red) in non-permeabilized cells. Each determination is the mean ± SD of two independent experiments performed in duplicate. Statistical analysis was performed with a *t* test. *, *P*<0.05; **, *P*<0.01 (compared with strain S2308). (**C**) Representative micrographs of J774.A1 macrophage-like cells infected for 0.5h (upper panel) and 24h (lower panel) with S2308 pGFP or *Δcgh* pGFP (MOI 10∶1). Insets show total bacteria in green and extracellular bacteria in red. Merged images show extracellular bacteria in green with a red outline, while intracellular bacteria are visualized only in green.

To assess whether the observed behavior is the consequence of a defect in adhesion and/or internalization, the percentage of macrophages with associated bacteria and the distribution of bacteria (intracellular or extracellular) per infected cell were examined by immunofluorescence microscopy at different times post-infection. As shown in Fig. 3A, at 0.5 h after infection 15.93±0.35% of macrophages were associated with wild type bacteria whereas 35.12±1.22% of macrophages were associated with large *Δcgh* aggregates (Fig. 3C), thus indicating that deletion of *cgh* affects the adherence of *B. abortus*. Even though the mutant had an increased adhesion capacity, its invasiveness was reduced. At 0.5 h after infection only 4.30±0.42% of *Δcgh* were internalized in comparison with 14.60±1.56% of intracellular wild type (Fig. 3B and C upper panel). At 24 h after infection, the majority of wild type bacteria were replicating within macrophages (72.90±0.57%) while the *Δcgh* mutant remained extracellular-associated forming bacterial aggregates, with only a small percentage (30.70±1.84%) intracellularly located (Fig. 3 B and C). Consistent with the CFU counts, at 48 h after infection 91.10±0.28% of the wild type were replicating compared to the 54.90±0.57% of the mutant. Altogether, these results demonstrate that the absence of CGH results in altered adhesion and internalization phenotypes.

Next, we analyzed the biogenesis of the Brucella-containing vacuole (BCV) by scoring the recruitment kinetics of the late-endosome/lysosome glycoprotein LAMP-1 and the endoplasmic reticulum membrane marker calnexin. There were no significant differences in the recruitment and subsequent exclusion of LAMP-1 to the wild type and *Δcgh* BCVs (Fig. S1). At 24 h p.i., the small fraction of internalized *Δcgh* was able to promote maturation of the replicative BCV and multiplied in calnexin-positive organelles as the wild type, a fact that explains the observed increase in the intracellular CFU from 24 h to 48 h p.i. (Fig. S2). Thus, the absence of CGH affects the adhesion and internalization of *B. abortus*, but does not affect the intracellular traffic and replication capacity.

### Cell envelope-associated proteins of Δcgh

The increased resistance to polycationic peptides as well as the defect in the internalization of Δ*cgh*, both indicative of surface alteration, led us to analyze the cell-envelope associated proteins of this mutant strain in search of differences with S2308. Representative proteome maps at three overlapping narrow pH ranges (i.e., 3.9 to 5.1, 4.7 to 5.9 and 5.5 to 6.7) of *B. abortus Δcgh* mutant strain are shown in Fig. S3. A total of 374 protein spots were detected. Of these, 92 protein spots were successfully identified by MALDI TOF MS, representing 59 ORFs (Table S1).

#### i. Overexpressed cell envelope-associated proteins of Δcgh

Cell envelope-associated proteins whose amounts increased 2.2 to 12.8-fold in *Δcgh* compared to their amounts in S2308 were categorized as overexpressed. A total of 32 overexpressed spots were identified by 2D PAGE and MALDI TOF MS (Table 1). Among these overexpressed proteins spots, 46.87% are predicted to be on the outer membrane, 28.12% to be cytoplasmic and periplasmic, while the subcellular location of the remaining 25.01% could not be determined. Computer-assisted analysis of average gels (composed of three replicate gels) revealed that several isoforms (mass and charge variants) of 25, 25c and 31 kDa outer membrane proteins (Omps) were significantly overexpressed in the mutant strain. Eleven isoforms of 25 kDa Omp and four isoforms of 31 kDa Omp were overexpressed as compared to their corresponding isoforms in S2308. Other overexpressed protein spots, already identified in the proteome analysis of *B. abortus* cell envelope [9], include aldehyde dehydrogenase, hypothetical protein BAB1_1489, peptidyl-prolyl *cis-trans* isomerase D, pyruvate dehydrogenase beta subunit, ATP synthase alpha and beta chains, cell division protein FtsZ, malate dehydrogenase, an isoform of the cell division inhibitor MinD and chaperonin protein DNAJ, among others.

**Table 1:** Overexpressed cell envelope-associated proteins in *B. abortus Δchg* as compared with S2308.

pH Range and Spot N°	Annotation	Accession Number	pI		MW (kDa)		Difference	ORF	Subcellular Location*
pH 3.9−5.1			Exp.	Theor.	Exp.	Theor.			
6	Hypothetical Protein	gi|17986850	4.96	11.26	74.4	18.2	9.8	BAB1_1867	C
8	Peptidyl –prolyl *cis-trans* isomerase D	gi|17987128	4.60	4.84	66.1	68.1	4.7	BAB1_1162	U
9	25 kDa Omp 28	gi|17987532	4.91	8.58	64.5	23.2	11.0	BAB1_0722	OM
12	Pyruvate dehydrogenase, beta subunit	gi|17987138	4.62	4.73	63.5	49.0	3.1	BAB1_1151	C
19	25 kDa Omp 7	gi|17987532	4.90	8.58	57.4	23.2	9.1	BAB1_0722	OM
26	31 kDa Omp 5	gi|17986685	4.65	5.04	32.3	22.0	12.8	BAB1_1639	OM
42	25 kDa Omp 24	gi|17987532	4.85	8.58	58.1	23.2	2.3	BAB1_0722	OM
47	25 kDa Omp	gi|17988112	4.92	4.78	25.3	24.6	2.5	BAB1_0116	OM
52	25 kDa Omp 3	gi|17988112	4.76	4.78	29.1	24.6	5.8	BAB1_0116	OM
55	Hypothetical Protein	gi|17986825	4.68	4.83	27.4	30.0	2.7	BAB1_1489	U
69	25 kDa Omp 15	gi|17987532	4.53	8.58	29.5	23.2	2.7	BAB1_0722	OM
107	25 kDa Omp 5	gi|17987532	4.63	8.58	66.6	23.2	4.0	BAB1_0722	OM
113	25 kDa Omp 18	gi|17987532	4.74	8.58	26.2	23.2	5.0	BAB1_0722	OM
121	31 kDa Omp 9	gi|17989189	4.86	5.21	64.2	23.3	6.8	BAB1_1639	OM
126	Aldehyde dehydrogenase	gi|17988023	4.40	5.99	31.4	51.1	2.8	BAB1_0211	C

pH 4.7–5.9									
10	25 kDa Omp 7	gi|3914203	5.15	8.58	55.4	23.2	2.1	BAB1_0722	OM
13	25 kDa Omp 4	gi|3914203	5.02	8.58	56.6	23.2	4.9	BAB1_0722	OM
19	25 kDa Omp 8	gi|3914203	5.12	8.58	43.6	23.2	4.0	BAB1_0722	OM
30	Malate dehydrogenase	gi|17986421	5.24	5.39	40.7	34.4	2.9	BAB1_1927	U
46	31 kDa Omp 4	gi|17989189	4.99	5.21	27.7	23.3	2.5	BAB1_1639	OM
52	31 kDa Omp 2	gi|17989189	4.84	5.21	26.8	23.3	4.0	BAB1_1639	OM
77	ATP synthase, beta subunit	gi|17986535	4.87	5.48	62.4	54.8	3.0	BAB2_0129	C
111	Cell division protein FtsZ	gi|23502296	5.39	5.34	83.2	60.7	3.6	BAB1_1444	U

pH 5.5–6.7									
10	NADP-specific glutamate dehydrogenase	gi|17988006	5.94	5.77	47.6	45.6	3.0	BAB1_0228	C
17	Putative glycerol-3-phosphate acyltransferase, PLSX	gi|17987464	6.03	6.03	47.6	36.9	2.7	BAB1_0797	C
20	TolB translocationprotein 2	gi|17986622	6.11	8.75	49.0	48.5	3.5	BAB1_1709	P
22	Glucose/ribitol dehydrogenase	gi|17986385	6.24	5.72	36.2	35.4	2.3	BAB1_1968	U
27	ATP synthasealpha chain 3	gi|17986533	6.42	6.1	64.3	54.9	2.2	BAB1_1809	U
38	Cell division inhibitor MinD	gi|17989271	6.32	5.42	29.3	27.1	5.7	BAB2_0883	C
39	3-ketoacyl-(acyl-carrier-protein) reductase	gi|17987760	6.44	6.44	26.7	26.0	3.1	BAB1_0483	C
51	Chaperone protein DNAJ	gi|17988284	6.34	6.51	42.6	41.1	2.3	BAB1_2130	C
59	Transcription antitermination protein NusG1	gi|17987027	5.99	6.64	20.9	19.7	2.6	BAB1_1269	U

Exp.: experimental; Theor.: theoretical; Omp: outer membrane protein;

*as determined by PSORTb version 3.0.2 [23]; OM: outer membrane; C: cytoplasmic; P: periplasmic; U: unknown.

#### ii. Underexpressed cell envelope-associated proteins of strain Δcgh

Several cell envelope-associated proteins of *Δcgh* were expressed at a significantly lower level (-2.0 to -27-fold) and thus were classified as underexpressed in comparison to their expression in S2308. A total of 39 underexpressed spots were identified by 2D PAGE and MALDI TOF MS (Table 2). Among these underexpressed proteins spots, 10.25% are predicted to be on the outer membrane, 69.23% to be cytoplasmic and periplasmic, while the subcellular location of the remaining 20.52% could not be determined. Several isoforms of four ribosomal proteins (large and small subunits) were underexpressed in *Δcgh* indicating a misregulation in the synthesis or possibly post-translational modifications of these proteins. Two isoforms of porin Omp2b, one of the major outer membrane proteins, were significantly underexpressed in the mutant strain. Other underexpressed proteins in the mutant strain, some of which have already been identified in the proteome analysis of *B. abortus* cell envelope [9], include three isoforms of 60 kDa chaperonin GroEL, two isoforms of termination factor Rho, an isoform of the cell division inhibitor MinD, DNA-directed RNA polymerase alpha chain, GroES chaperonin, 22 kDa Omp, ATP synthase alpha and beta chains, glyceraldehyde-3-phosphate dehydrogenase and tetratricopeptide repeat family protein, among others.

**Table 2:** Underexpressed cell envelope-associated proteins in *B. abortus Δchg* as compared with S2308.

pH Range and Spot N°	Annotation	Accession Number	pI		MW (kDa)		Difference	ORF	Subcellular Location*
pH 3.9–5.1			Exp.	Theor.	Exp.	Theor.			
24	Omp2b porin 7	gi|17987588	4.38	4.61	45.1	40.4	−18.6	BAB1_0660	OM
75	Omp2b porin 6	gi|17987588	4.41	4.61	40.7	40.4	−12.5	BAB1_0660	OM
98	LSU ribosomal protein L9P 2	gi|17987766	4.01	4.79	20.6	20.9	−4.7	BAB1_0477	C
100	LSU ribosomal protein L12P	gi|17987031	4.70	4.79	13.1	12.5	−3.0	BAB1_1265	U

pH 4.7–5.9									
3	60 kDa chaperonin GroEL 2	gi|17989393	5.03	5.04	72.5	57.5	−3.1	BAB2_0189	C
7	SSU ribosomal protein S1P	gi|17988198	5.09	5.19	78.3	63.6	−5.2	BAB1_0025	C
8	SSU ribosomal protein S1P 4	gi|17988198	5.07	5.19	80.1	63.6	−6.7	BAB1_0025	C
15	60 kDa chaperonin GroEL 9	gi|17989393	5.04	5.04	49.9	57.5	−2.7	BAB2_0189	C
21	DNA-directed RNA polymerase alpha chain	gi|17987064	4.95	4.85	47.3	37.3	−2.2	BAB1_1231	C
28	60 kDa chaperonin GroEL 3	gi|17989393	4.98	5.04	62.6	57.5	−2.0	BAB2_0189	C
29	ATP synthase beta chain 3	gi|17986535	5.25	5.48	56.9	54.8	−2.5	BAB1_1807	U
36	Hypothetical protein	gi|17986886	5.23	5.18	25.9	21.0	−2.3	BAB1_1423	C
37	Hypothetical protein 1	gi|17987581	5.24	5.80	23.4	22.1	−6.1	BAB1_0669	C
42	LSU ribosomal protein L9P 2	gi|17987766	4.90	4.79	20.6	20.9	−5.6	BAB1_0477	C
44	LSU ribosomal protein L9P	gi|17987766	4.94	4.79	20.0	20.9	−3.5	BAB1_0477	C
45	LSU ribosomal protein L9P 1	gi|17987766	4.97	4.79	20.1	20.9	−3.4	BAB1_0477	C
55	DNA gyrase subunit B	gi|17986651	5.11	5.11	11.2	12.7	−3.1	BAB1_1675	U
62	SSU ribosomal protein S1P 1	gi|17988198	5.13	5.19	78.1	63.6	−5.0	BAB1_0025	C
63	SSU ribosomal protein S1P 2	gi|17988198	5.16	5.19	78.1	63.6	−3.2	BAB1_0025	C
65	DegT/DnrJ/EryC1/StrS aminotransferase	gi|17986704	5.24	5.50	48.7	41.8	−3.8	BAB1_1616	U
69	3-demethylubiquinone-9 3-methyltransferase	gi|17986472	5.54	5.79	29.2	27.5	−4.7	BAB1_1875	C
70	Cell division inhibitor MinD	gi|17989271	5.49	5.42	27.7	27.1	−8.6	BAB2_0883	C
80	LSU ribosomal protein L25P	gi|17986764	5.54	5.92	24.8	25.1	−5.2	BAB1_1551	C
84	Co-chaperonin GroES	gi|62317143	5.06	5.41	11.5	10.4	−5.7	BAB2_0190	C
86	Hypothetical protein	gi|17987375	5.01	4.53	28.1	17.6	−5.4	BAB1_0893	U
97	Surface antigen	gi|17988178	5.35	5.57	82.8	67.2	−6.0	BAB1_0045	OM
106	Succinyl-CoA synthetasealpha chain	gi|17986423	5.49	5.81	34.3	31.2	−3.2	BAB1_1925	C
109	Aspartyl/glutamyl-tRNA amidotransferase subunit A	gi|39931612	5.41	5.57	60.3	52.5	−2.1	BAB2_0646	C

pH 5.5–6.7									
3	Transcription termination factor Rho	gi|17986287	5.76	5.87	57.3	47.0	−5.1	BAB1_2065	C
4	Transcription termination factor Rho 1	gi|17986287	5.89	5.87	56.3	47.0	−2.3	BAB1_2065	C
6	2-octaprenyl-3-methyl-6-methoxy-1,4-benzoquinol hydroxylase	gi|17986449	5.96	6.29	53.3	46.6	−4.7	BAB1_1898	C
11	UDP-3-O-hydroxymyristol glucosamine N-acyltransferase	gi|17987114	5.97	6.00	43.8	36.4	−2.1	BAB1_1175	U
16	Biphenyl-2,3-diol 1,2-dioxygenase III	gi|17984445	6.05	6.11	28.8	30.0	−26.9	BAB2_0231	C
25	Gliceraldehyde-3-phosphate dehydrogenase	gi|17986593	6.22	6.13	47.2	36.3	−2.8	BAB1_1741	C
31	3-ketoacyl-(acyl-carrier-protein) reductase	gi|17987760	6.25	6.44	25.0	26.0	−6.7	BAB1_0483	C
32	22 kDa Omp	gi|17987000	6.19	8.59	23.3	19.4	−2.7	BAB1_1302	OM
45	Argininosuccinate synthase	gi|17988153	6.15	6.06	57.9	45.3	−4.5	BAB1_0071	C
48	ATP synthase alpha chain 4	gi|17986533	5.92	6.10	63.1	54.9	−2.8	BAB1_1809	U
66	Tetratricopeptide repeat family protein	gi|17987814	5.74	5.75	76.5	66.5	−2.6	BAB1_0430	P

Exp.: experimental; Theor.: theoretical; Omp: outer membrane protein;

*as determined by PSORTb version 3.0.2 [23]; OM: outer membrane; C: cytoplasmic; P: periplasmic; U: unknown.

#### iii. Unique cell envelope-associated proteins of strain Δcgh

Unique spots are mostly protein isoforms that are not encoded by unique ORFs. However, proteins MW and pI are unique probably due to post-translational modifications. A majority of these protein spots are predicted to be on the outer membrane. Some of these unique spots are therefore charge and mass variants of single ORFs. Six variants of 31 kDa Omp and 4 variants of 25 kDa Omp were unique to *Δcgh* (Table 3). Other unique spots include ATP synthase beta chain, chaperonin ClpA/B and chromosome partitioning protein PARB.

**Table 3:** Unique cell envelope-associated proteins in *B. abortus Δchg* as compared with S2308.

pH Range and Spot N°	Annotation	Accession Number	pI		MW (kDa)		ORF	Subcellular Location*
pH 3.9–5.1			Exp.	Theor.	Exp.	Theor.		
144	25 kDa Omp 10	gi|17987290	4.25	4.72	30.5	25.2	BAB1_0116	OM
162	25 kDa Omp 21	gi|17987532	4.95	8.58	47.2	23.2	BAB1_0722	OM
167	25 kDa Omp 9	gi|17987290	4.22	4.72	33.9	25.2	BAB1_0116	OM
196	Surface antigen	gi|1262291	4.31	8.40	32.9	86.5	BAB1_1176	OM
197	31 kDa Omp 6	gi|17986685	4.90	5.04	28.9	22.0	BAB1_1639	OM
198	25 kDa Omp 6	gi|17987532	4.66	8.58	65.8	23.2	BAB1_0722	OM
199	ND		4.54		64.9			
200	ATP synthase beta chain	gi|17986535	4.56	5.48	66.7	54.8	BAB1_1807	U
201	31 kDa Omp 11	gi|17986685	4.47	5.04	31.7	22.0	BAB1_1639	OM
217	ND		4.81		65.5			
219	ND		4.59		33.8			
220	ND		4.56		34.8			
221	ND		4.69		68.9			
222	ND		4.60		48.0			

pH 4.7–5.9								
131	31 kDa Omp 7	gi|17989189	4.94	5.21	61.8	23.3	BAB1_1639	OM
147	Surface antigen	gi|17987113	5.14	5.37	96.0	85.9	BAB1_1176	OM
189	Chaperonin ClpA/B	gi|23502870	5.27	5.40	39.8	36.7	BAB1_2023	C
190	31 kDa Omp 3	gi|17989189	4.92	5.21	29.9	23.3	BAB1_1639	OM
191	31 kDa Omp 5	gi|17989189	5.07	5.21	27.7	23.3	BAB1_1639	OM
198	31 kDa Omp 8	gi|17989189	5.01	5.21	62.0	23.3	BAB1_1639	OM
216	ND		5.01		25.8			

pH 5.5–6.7								
85	ND		5.66		80.6			
88	Chromosome partitioning protein PARB	gi|17986294	6.11	5.81	36.1	32.6	BAB1_2059	C

ND: not determined; Exp.: experimental; Theor.: theoretical; Omp: outer membrane protein;

*as determined by PSORTb version 3.0.2 [23]; OM: outer membrane; C: cytoplasmic; U: unknown.

### Expression of some major outer membrane proteins is altered in B. abortus Δcgh

**Figure 4:**
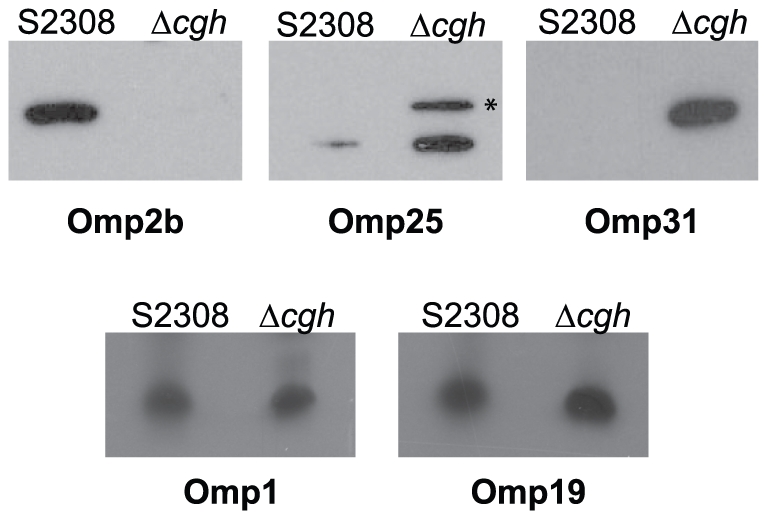
*B. abortus Δcgh* shows an altered expression profile of some major Omps. Protein levels of Omp 2b, Omp25, Omp25c (*), Omp31b, Omp1 and Omp19 in wild-type *B. abortus* (S2308) and its isogenic deletion mutant *Δcgh* were determined by Western blot analysis with monoclonal antibodies.

The *Brucella* Omp25/Omp31 family is composed of seven homologous Omps: Omp25, Omp25b, Omp25c, Omp25d, Omp31 and Omp31b [11], [12]. In order to confirm the composition of the cell envelope-associated proteome, we analyzed the expression of four Omps in the wild type and the *Δcgh* mutant strains. Protein expression levels of Omp2b (BAB1_0660), Omp25 (BAB1_0722), Omp25c (BAB1_0116) and Omp31b (BAB1_1639) were determined by Western blot analysis using monoclonal antibodies against these proteins. Protein levels of Omp1 and Omp19 were also determined to standardize the protein load. In agreement with the results obtained in the proteomic analysis, we did not detect expression of Omp2b in *Δcgh* (Fig. 4). Higher levels of Omp25 and Omp25c were detected in *Δcgh* when compared with S2308, and expression of Omp31b was only detected in the mutant (Fig. 4). These results confirm proteomic data of cell envelope-associated proteins and provide evidence of an overall alteration of *Δcgh's* cell envelope composition. This finding could be related to the mutant's impaired ability to be internalized into host cells as well as its increased resistance to polymyxin B.

## Discussion

In a previous study, *B. abortus* CGH was shown to cleave glycocholate into glycine and cholate [2] and this activity was essential for *B. abortus* resistance to bile salts. These findings, together with the fact that a *cgh*-deletion mutant is attenuated in mice inoculated through the oral route, are in clear agreement with CGH role in *B. abortus* resistance to the action of these biological detergents. However, attenuation of the mutant strain in intraperitoneally inoculated mice suggested that CGH could play another relevant role for infection through the systemic route [2]. CGH, together with penicillin acylase and acid ceramidase, belong to the family of linear amide C-N hydrolases (Pfam 02275). Members of this family, which belong to the N-terminal nucleophil (Ntn) hydrolase superfamily, catalyze the hydrolysis of amide bonds, other than peptide bonds, in linear amides present in proteins, peptidoglycan or small molecules, raising the possibility that *B. abortus* CGH could hydrolyze amide bonds on substrates other than host's bile salts.

In this work, we confirmed *B. abortus* CGH participation in bile salts resistance and presented additional evidence indicating that the enzyme is important for maintaining the composition and properties of the *Brucella* cell envelope.

We found an increased resistance of *Δcgh* mutant to the bactericidal action of polymyxin B as well as increased growth capability at both neutral and acidic pH. The requirement of *B. abortus* CGH activity during the early phases of infection was demonstrated both in phagocytic and non-phagocytic cells. It was shown that *Δcgh* has an altered adhesion and internalization behavior. The mutant exhibits an increased adhesion to the host-cell, forming bacterial aggregates not observed in the parental wild type strain and shows a significant delay in the internalization process. At 48 h after infection, about 45% of *Δcgh* were found associated to macrophages in extracellular clumps (Fig. 3). However, a small fraction of the mutant was able to invade and promote the maturation of the replicative LAMP1-negative, Calnexin-positive BCV where bacterial replication occurred even at a faster intracellular growth rate (Fig. 2 and Fig. S1 and S2). These results suggested altered outer membrane properties and composition of the mutant strain.

To evaluate this hypothesis, a proteomic analysis of cell envelope-associated proteins was performed in order to compare the membrane composition of *Δcgh* mutant and wild-type *B. abortus* S2308. Among the observed differences, the most important differential expression profiles involved four major Omps: several isoforms of 25 kDa and 31 kDa Omps were significantly overexpressed in *Δcgh* while two isoforms of porin 2b were significantly underexpressed in the mutant strain. The opposite scenario was described in a previous study when the outer membrane proteome of a *B. melitensis virB* mutant was analyzed. *virB* genes code for a Type IV Secretion System (VirB), a membrane–associated multiprotein complex essential for *Brucella* intracellular survival and replication [13]. Proteomic analysis of a *B. melitensis virB* mutant showed reduced expression of several members of the Omp25/Omp31 family [14]. Interestingly, this mutant was more sensitive to polymyxin B, suggesting a role for Omp25/Omp31 members in resistance to this cationic detergent. In addition, the strong association of some of the members of this family with LPS suggests that they play an important structural role in the outer membrane [11], [15], which may be central to the interactions between *Brucella* and the host cell. Regarding Omp2b, underexpression of this Omp may be the result of a compensatory mechanism necessary to restore the balance essential for the stability of the outer membrane [16]. Additionally, the tubulin-like protein FtsZ and MinD, involved in Z ring formation and inhibition of Z ring polymerization, respectively, showed altered membrane expression in *Δcgh*, which may be related to the increased growth performance of the mutant either during vegetative and intracellular stages.

Overall, we have shown that *B. abortus Δcgh* has several pleiotropic effects characterized by resistance to polymyxin B, reduced generation time, altered adhesion and internalization into host-cell and modified cell envelope protein composition, all features indicating that CGH plays an important function in maintaining the *Brucella* outer membrane architecture. Since *B. abortus* CGH sequence contains a putative signal sequence [2], it is probable that the enzyme exerts its action on substrates located either in the cell envelope or in the extracellular medium. Moreover, recent studies identified *B. abortus* CGH as an extracellular protein released to the growth medium [17] and as an abundant component of the BCV [5], two findings that support extracellular activity of CGH.

Apart from enhancing *Brucella* survival during its transit through the host's gut, acting as bile salt deconjugating enzyme, the phenotypes described in this work point to a role of CGH in maintaining the cell envelope structure. Further investigation of *B. abortus* CGH activity, including the identification of its substrates and understanding how the activity affects overall cell envelope composition, will provide insight into the importance of the outer membrane architecture for the *Brucella* pathogenesis.

## Supporting Information

Figure S1 **LAMP-1 acquisition.** Representative images of LAMP-1 acquisition by BCVs in J774.A1 macrophage-like cells infected with *B. abortus* S2308 (WT) (**A**) or *Δcgh* mutant strain (**B**) at 4 h p.i. Insets show intracellular *Brucella* within LAMP-1 positive vacuoles. (**C**) Quantification of LAMP-1 acquisition by BCVs in J774.A1 macrophage-like cells infected with *B. abortus* S2308 (WT) or *Δcgh* mutant strain at 4 and 24 h p.i. Data are means of two independent experiments.
(EPS)

Figure S2 **Calnexin acquisition.** Representative images of calnexin acquisition by BCVs in J774.A1 macrophage-like cells infected with *B. abortus* S2308 (WT) (**A**) or *Δcgh* mutant strain (**B**) at 24 h p.i. Insets show intracellular *Brucella* within calnexin positive vacuoles.
(EPS)

Figure S3 **Membrane proteome maps of laboratory grown** ***B. abortus Δcgh*** **in the pH ranges of 3.9 to 5.1 (A), 4.7 to 5.9 (B) and 5.5 to 6.7 (C).** Membrane enriched fractions (30 µg) were focused with IPG strips and run on 6–15% gradient SDS-PAGE. The gels were stained with SYPRO® Ruby and imaged at 470 nm. Protein spots successfully identified by MALDI-TOF MS are listed in Table S1.
(TIF)

Table S1 **Identified cell envelope-associated proteins in** ***B. abortus Δcgh*** **as determined by 2-DE and MALDI-TOF MS.**
(DOC)
