# Supplementary figures and images for: Brucella abortus Choloylglycine Hydrolase Affects Cell Envelope Composition and Host Cell Internalization
Source: PLoS One. 2011 Dec 8;6(12):e28480. doi: 10.1371/journal.pone.0028480 (PMC3234258; doi:10.1371/journal.pone.0028480)

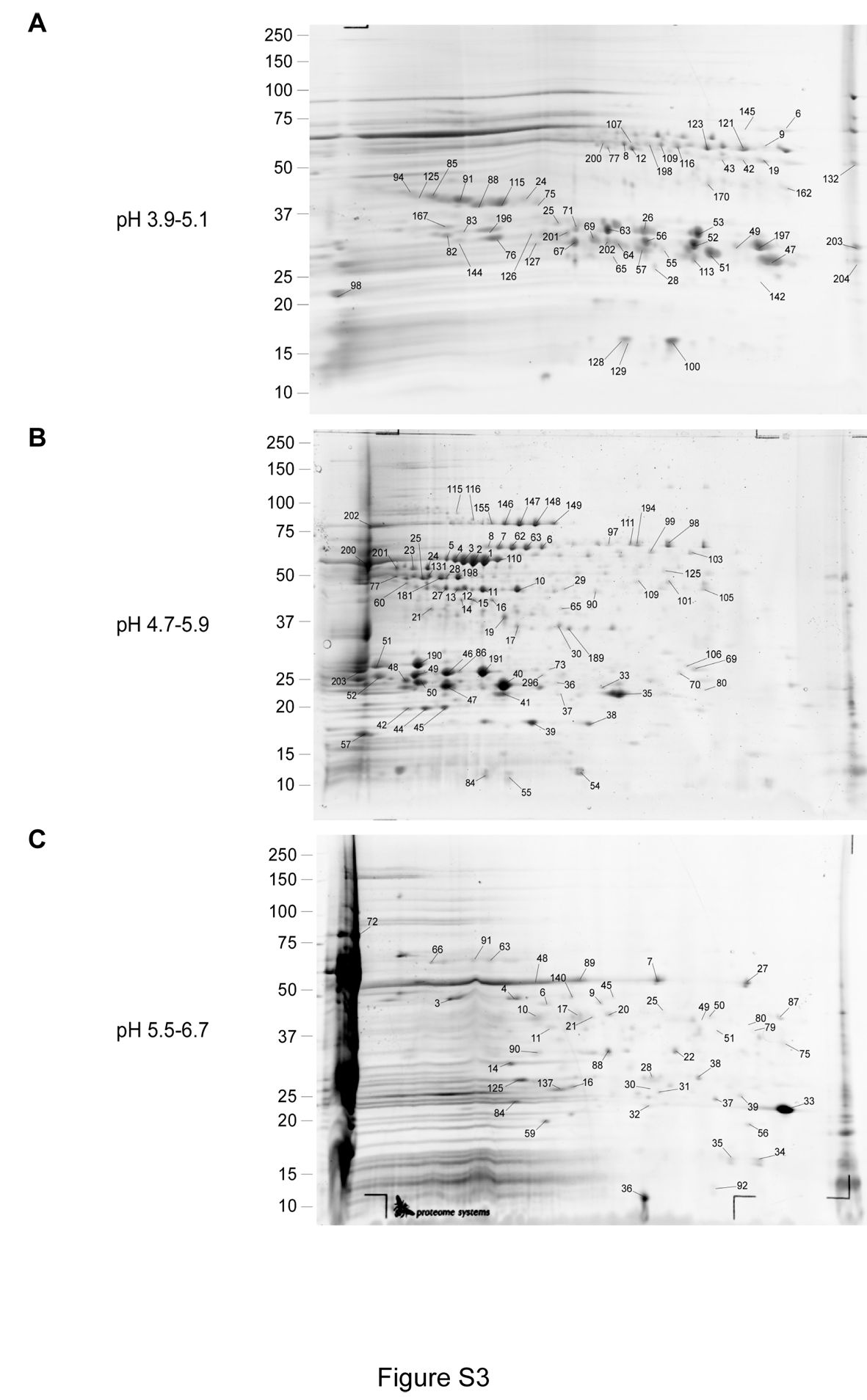

Supplement: Figure S3 — Membrane proteome maps of laboratory grown B. abortus Δcgh in the pH ranges of 3.9 to 5.1 (A), 4.7 to 5.9 (B) and 5.5 to 6.7 (C). Membrane enriched fractions (30 µg) were focused with IPG strips and run on 6–15% gradient SDS-PAGE. The gels were stained with SYPRO® Ruby and imaged at 470 nm. Protein spots successfully identified by MALDI-TOF MS are listed in Table S1. (TIF) [file pone.0028480.s003.tif]
