# Supplementary material for: Brucella abortus Choloylglycine Hydrolase Affects Cell Envelope Composition and Host Cell Internalization
Source: PLoS One. 2011 Dec 8;6(12):e28480. doi: 10.1371/journal.pone.0028480 (PMC3234258; doi:10.1371/journal.pone.0028480)
Supplement: Table S1 — Identified cell envelope-associated proteins in B. abortus Δcgh as determined by 2-DE and MALDI-TOF MS. (DOC) [file pone.0028480.s004.doc]

**TABLE S1.** Identified cell envelope-associated proteins in *B. abortus Δcgh* as determined by 2-DE and MALDI-TOF MS.

| **pH Range and Spot N°** | **Annotation** | **Accession Number** | **ORF** | **Subcellular**  **Location*** |
| --- | --- | --- | --- | --- |
| **pH 3.9-5.1** |  |  |  |  |
| 1 | Hypothetical protein | gi|17987892 | BAB1_0343 | OM |
| 6 | Hypothetical Protein | gi|17986850 | BAB1_1867 | C |
| 8 | Peptidyl-prolyl *cis-trans* isomerase D | gi|17987128 | BAB1_1162 | U |
| 12 | Pyruvate dehydrogenase, beta subunit | gi|17987138 | BAB1_1151 | C |
| 28 | Hypothetical protein BAB1_1489 a | gi|17986825 | BAB1_1489 | U |
| 43 | Hypothetical protein | gi|23502737 | BAB1_1885 | U |
| 47 | 25 kDa Omp-a | gi|17988112 | BAB1_0116 | OM |
| 49 | 31 kDa Omp-a | gi|17986685 | BAB1_1639 | OM |
| 51 | 25 kDa Omp-b | gi|17988112 | BAB1_0116 | OM |
| 53 | 31 kDa Omp-b | gi|17986685 | BAB1_1639 | OM |
| 55 | Hypothetical protein BAB1_1489 | gi|17986825 | BAB1_1489 | U |
| 56 | 31 kDa Omp-c | gi|17986685 | BAB1_1639 | OM |
| 63 | 31 kDa Omp-d | gi|17986685 | BAB1_1639 | OM |
| 65 | OmpW family outer membrane protein | gi|17986737 | BAB1_1579 | OM |
| 71 | 31 kDa Omp-e | gi|17986685 | BAB1_1639 | OM |
| 75 | Omp2b Porin-a | gi|17987588 | BAB1_0660 | OM |
| 88 | Omp2b Porin-b | gi|17987588 | BAB1_0660 | OM |
| 91 | Omp2b Porin-c | gi|17987588 | BAB1_0660 | OM |
| 98 | LSU ribosomal protein L9P | gi|17987766 | BAB1_0477 | C |
| 100 | LSU ribosomal protein L12P | gi|17987031 | BAB1_1265 | U |
| 115 | Omp2b Porin-e | gi|17987588 | BAB1_0660 | OM |
| 123 | Trigger factor PPIASE | gi|17987352 | BAB1_0917 | U |
| 126 | Aldehyde dehydrogenase | gi|17988023 | BAB1_0211 | C |
| 129 | 25kDa Omp-c | gi|17988112 | BAB1_0116 | OM |
| 196 | Surface antigen | gi|1262291 | BAB1_1176 | OM |
| **pH 4.7-5.9** |  |  |  |  |
| 1 | 60kDa chaperonin GroEL-a | gi|17989393 | BAB2_0189 | C |
| 2 | 60kDa chaperonin GroEL-b | gi|17989393 | BAB2_0189 | C |
| 3 | 60kDa chaperonin GroEL-c | gi|17989393 | BAB2_0189 | C |
| 7 | SSU ribosomal protein S1P-a | gi|17988198 | BAB1_0025 | C |
| 14 | Enolase-a | gi|17987134 | BAB1_1155 | C |
| 15 | Enolase-b | gi|17987134 | BAB1_1155 | C |
| 16 | ATP synthase beta subunit-a | gi|17986535 | BAB2_0129 | C |
| 21 | DNA-directed RNA polymerase alpha chain | gi|17987064 | BAB1_1231 | C |
| 24 | 60kDa chaperonin GroEL-d | gi|17989393 | BAB2_0189 | C |
| 25 | 60kDa chaperonin GroEL-e | gi|17989393 | BAB2_0189 | C |
| 28 | 60kDa chaperonin GroEL-f | gi|17989393 | BAB2_0189 | C |
| 29 | ATP synthase beta subunit-b | gi|17986535 | BAB1_1807 | U |
| 30 | Malate dehydrogenase | gi|17986421 | BAB1_1927 | U |
| 33 | Hypothetical protein-a | gi|17987581 | BAB1_0669 | C |
| 35 | Transaldolase | gi|17986528 | BAB1_1813 | C |
| 36 | Hypothetical protein | gi|17986886 | BAB1_1423 | C |
| 37 | Hypothetical protein-b | gi|17987581 | BAB1_0669 | C |
| 38 | DNA protection during starvation protein-a | gi|17988263 | BAB1_2150 | C |
| 39 | DNA protection during starvation protein-b | gi|17988263 | BAB1_2150 | C |
| 40 | 25 kDa Omp | gi|17987532 | BAB1_0722 | OM |
| 44 | LSU ribosomal protein L9P-a | gi|17987766 | BAB1_0477 | C |
| 45 | LSU ribosomal protein L9P-b | gi|17987766 | BAB1_0477 | C |
| 49 | 31 kDa Omp-a | gi|17986685 | BAB1_1639 | OM |
| 51 | 31 kDa Omp-b | gi|17986685 | BAB1_1639 | OM |
| 54 | Co-chaperonin GroES | gi|62317143 | BAB2_0190 | C |
| 55 | DNA gyrase subunit B | gi|17986651 | BAB1_1675 | U |
| 60 | 60kDa chaperonin GroEL-g | gi|17989393 | BAB2_0189 | C |
| 62 | SSU ribosomal protein S1P-b | gi|17988198 | BAB1_0025 | C |
| 63 | SSU ribosomal protein S1P-c | gi|17988198 | BAB1_0025 | C |
| 65 | DegT/DnrJ/EryC1/StrS aminotransferase | gi|17986704 | BAB1_1616 | U |
| 69 | 3-demethylubiquinone-9 3-methyltransferase -b | gi|17986472 | BAB1_1875 | C |
| 76 | 60kDa chaperonin GroEL-h | gi|17989393 | BAB2_0189 | C |
| 77 | ATP synthase beta subunit-d | gi|17986535 | BAB2_0129 | C |
| 80 | LSU ribosomal protein L25P | gi|17986764 | BAB1_1551 | C |
| 86 | Hypothetical protein | gi|17987375 | BAB1_0893 | U |
| 90 | Adenosylhomocysteinase | gi|17988312 | BAB1_2099 | C |
| 98 | Surface antigen | gi|17988178 | BAB1_0045 | OM |
| 99 | Metallopeptidase family M24 | gi|17986874 | BAB1_1437 | C |
| 100 | Cell division protein FtsZ-a | gi|17986868 | BAB1_1444 | U |
| 101 | Transcription termination factor rho-a | gi|17986287 | BAB1_2065 | C |
| 103 | Tetratricopeptide repeat family protein | gi|17987814 | BAB1_0430 | U |
| 105 | Transcription termination factor rho-b | gi|17986287 | BAB1_2065 | C |
| 106 | Succinyl-CoA synthetase alpha chain | gi|17986423 | BAB1_1925 | C |
| 109 | Aspartyl/glutamyl-tRNA amidotransferase subunit A | gi|39931612 | BAB2_0646 | C |
| 111 | Cell division protein FtsZ-b | gi|23502296 | BAB1_1444 | U |
| **pH 5.5-6.7** |  |  |  |  |
| 3 | Transcription termination factor rho-a | gi|17986287 | BAB1_2065 | C |
| 4 | Transcription termination factor rho-b | gi|17986287 | BAB1_2065 | C |
| 6 | 2-octaprenyl-3-methyl-6-methoxy-1,4-benzoquinol hydroxylase | gi|17986449 | BAB1_1898 | C |
| 10 | NADP-specific glutamate dehydrogenase | gi|17988006 | BAB1_0228 | C |
| 11 | UDP-3-O-hydroxymyristol glucosamine N-acyltransferase | gi|17987114 | BAB1_1175 | U |
| 15 | 3-demethylubiquinone-9 3-methyltransferase | gi|17986472 | BAB1_1875 | C |
| 16 | Biphenyl-2,3-diol 1,2-dioxygenase III | gi|17984445 | BAB2_0231 | C |
| 17 | Putative glycerol-3-phosphate acyltransferase, PLSX | gi|17987464 | BAB1_0797 | C |
| 22 | Glucose/ribitol dehydrogenase-b | gi|17986385 | BAB1_1968 | U |
| 25 | Gliceraldehyde-3-phosphate dehydrogenase | gi|17986593 | BAB1_1741 | C |
| 27 | ATP synthase alpha chain 3 | gi|17986533 | BAB1_1809 | U |
| 32 | 22 kDa Omp | gi|17987000 | BAB1_1302 | OM |
| 34 | SSU ribosomal protein S6P | gi|17987763 | BAB1_0480 | C |
| 35 | Acetyltransferase | gi|17986442 | BAB1_1904 | U |
| 36 | Membrane-bound proton-translocating pyrophsphatase | gi|17987088 | BAB1_0793 | U |
| 38 | Cell division inhibitor MinD | gi|17989271 | BAB2_0883 | C |
| 39 | 3-ketoacyl-(acyl-carrier-protein) reductase | gi|17987760 | BAB1_0483 | C |
| 45 | Argininosuccinate synthase | gi|17988153 | BAB1_0071 | C |
| 49 | TolB translocation protein | gi|17986622 | BAB1_1709 | P |
| 51 | Chaperone protein DNAJ | gi|17988284 | BAB1_2130 | C |
| 56 | Transcription antitermination protein NusG-a | gi|17987027 | BAB1_1269 | U |
| 88 | Chromosome partitioning protein PARB | gi|17986294 | BAB1_2059 | C |

Omp: outer membrane protein; * as determined by PSORTb version 3.0.2 [23]; OM: outer membrane; C: cytoplasmic; P: periplasmic; U: unknown.
